# Transgenerational effects of prenatal restricted diet on gene expression and histone modifications in the rat

**DOI:** 10.1371/journal.pone.0193464

**Published:** 2018-02-23

**Authors:** Joanna Nowacka-Woszuk, Izabela Szczerbal, Anna M. Malinowska, Agata Chmurzynska

**Affiliations:** 1 Department of Genetics and Animal Breeding, Poznan University of Life Sciences, Wolynska 33, Poznan, Poland; 2 Institute of Human Nutrition and Dietetics, Poznan University of Life Sciences, Wojska Polskiego 31, Poznan, Poland; Ludwig-Maximilians-Universitat Munchen Adolf-Butenandt-Institut, GERMANY

## Abstract

Dietary triggers acting on a developing fetus can affect the functioning of the body in later life; this can be observed on various levels, including epigenetic modifications and gene expression. Early-life programmed changes may be transmitted to successive generations. In this study, the impact of prenatal restricted diet was studied in four generations of rats. We hypothesized that this diet can induce changes in the expression of major genes involved in two epigenetic mechanisms: DNA methylation and histone modifications. The transcript level of six genes involved in these processes (*Dnmt1*, *Dnmt3a*, *Dnmt3b*, *Mecp2*, *Hdac1*, and *Sin3a*) was therefore determined in three tissues (liver, adipose, and muscle). This diet was found to have no effect on the F0 pregnant females. In the F1 progeny (fetuses at day 19 of pregnancy and 4-week-old rats) significant differences in the expression of the genes were observed mostly in the liver; in subsequent generations, we therefore studied only this tissue. Among the genes encoding DNA methyltransferases, significant changes were observed for *Dnmt1* in the F1 animals from the restricted group, but these were no longer evident in F2 and F3. The *Dnmt3a* and *Dnmt3b* genes showed no differences in mRNA level in F1 fetuses. Concerning the transcript level of the *Mecp2* gene only in F1 generation significant changes were found. For the histone modification genes, an increase in the expression of *Hdac1* in fetus liver was found in F1 and F2, while its level decreased in F3. The abundance of the *Sin3a* transcript varied in all generations. It was also found that the mRNA levels of the studied genes correlated highly positive with each other, but only in fetuses from the F1 restricted group. The DNA methylation cell potential, defined as the ratio of SAM (S-adenosylmethionine) to SAH (S-adenosylhomocysteine), was measured in the liver, with no alterations being found in the restricted groups. Evaluation of global histone H3 acetylation showed that it underwent a significant increase in the fetal livers of F1, while during aging (four-week old animals) this difference was no longer maintained. A tendency of increased H3 acetylation in fetuses was also detected in F2 generation. In F1 fetuses from restricted group the increased H3 acetylation positively correlated with transcriptional status of the studied genes. Our results indicate that the prenatal restriction diet can affect the activity of genes involved in epigenetic mechanisms in the liver across generations. Moreover, this feeding type influenced the global histone H3 acetylation in fetal liver.

## Introduction

The phenomenon of fetal programming occurs when environmental factors such as diet, age, disease, pharmacological treatment, and behavior act on pregnant dams to induce changes in the functioning of progeny organism; these changes manifest as differences in gene expression, protein activity, epigenetic marks, and metabolism [1]. Different feeding approaches have been tested for their programming potential, and insufficient nutrient supplies and total restricted diets during pregnancy have already been studied in rodent models. It has been shown that prenatal undernutrition may have long-term consequences, including increased susceptibility to excessive adiposity [2], insulin resistance [3], and changes in lipid metabolism [4]. These alterations in metabolism and physiology result from changed gene expression patterns [5]. Liver tissue has mainly been examined in studies of lipid and carbohydrate metabolism, though in fact programming may affect different tissues to different extents. For that reason, the effect of fetal nutrition should be examined in all tissues involved in the biological process under scrutiny. Recently, it has been postulated that different dietary regimens may permanently affect phenotype through epigenetic mechanisms; DNA methylation has been the most extensively studied of these [1, 6]. Differences in hepatic global DNA methylation in murine fetuses following maternal food restriction were described by Ogawa et al. [7]. They found that gestational undernutrition led to changes in the DNA methylation profile of many genes involved in insulin resistance, cholesterol and fatty acid metabolism, and immune system response. In the work of Ganguly et al. [8], locus-specific DNA methylation differences were seen in response to maternal calorie-restricted diets in mice, but they also noticed changes in protein-binding force (e.g. Mecp2 and Hdac2) to CpG islands, what could consequently induce alterations in other epigenetic mechanisms, such as chromatin conformation and histone modifications.

It is well known that, once programmed, changes during early life do not only directly affect the organism, but can also be transmitted to subsequent generations [9, 10]. Environmental factors acting on pregnant dams (F0) can induce direct changes in these animals, but also can indirectly act on the fetus (F1), as well as on the F1 germ cells which will generate the F2 animals. Thus, F3 is the first generation that is not exposed to the factor [11]. Transgenerational inheritance has been observed for different factors, including air pollution, nutrition, drugs, physical activity, radiation, and others. Lupu et al. [12] have reviewed the effect of various triggers that can affect metabolism, behavior, fertility, and hormonal setup through differences in gene expression and changes in epigenetic marks. The alterations they observed were inherited by subsequent generations. Concerning feeding type, a protein-deficient diet studied by Burdge et al. [13] had an effect on the methylation of specific gene promoters. These changes were found in the progeny from the F1 and F2 generations. In the study of Ponzio et al. [10], a calorie-restricted diet was applied to rats during gestation and examined in terms of blood pressure and vasodilatory response to acetylcholine in the adult progeny. The results indicated that the changes were noted in the male F1 offspring, and were also significant in the F2 and F3 generations.

In this study, we hypothesized that a restricted diet during rat pregnancy can induce changes in the expression of genes associated with epigenetic mechanisms, and that these alterations are inherited by subsequent generations. To verify this, the cell DNA methylation potential was estimated by measuring S-adenosylmethionine and S-adenosylhomocysteine levels, and the expression profiles of the key genes involved in the process were studied. Moreover, the global histone H3 acetylation and mRNA level of genes encoding enzymes associated with chromatin modification machinery were evaluated.

## Material and methods

### Animals and diets

**Fig 1:**
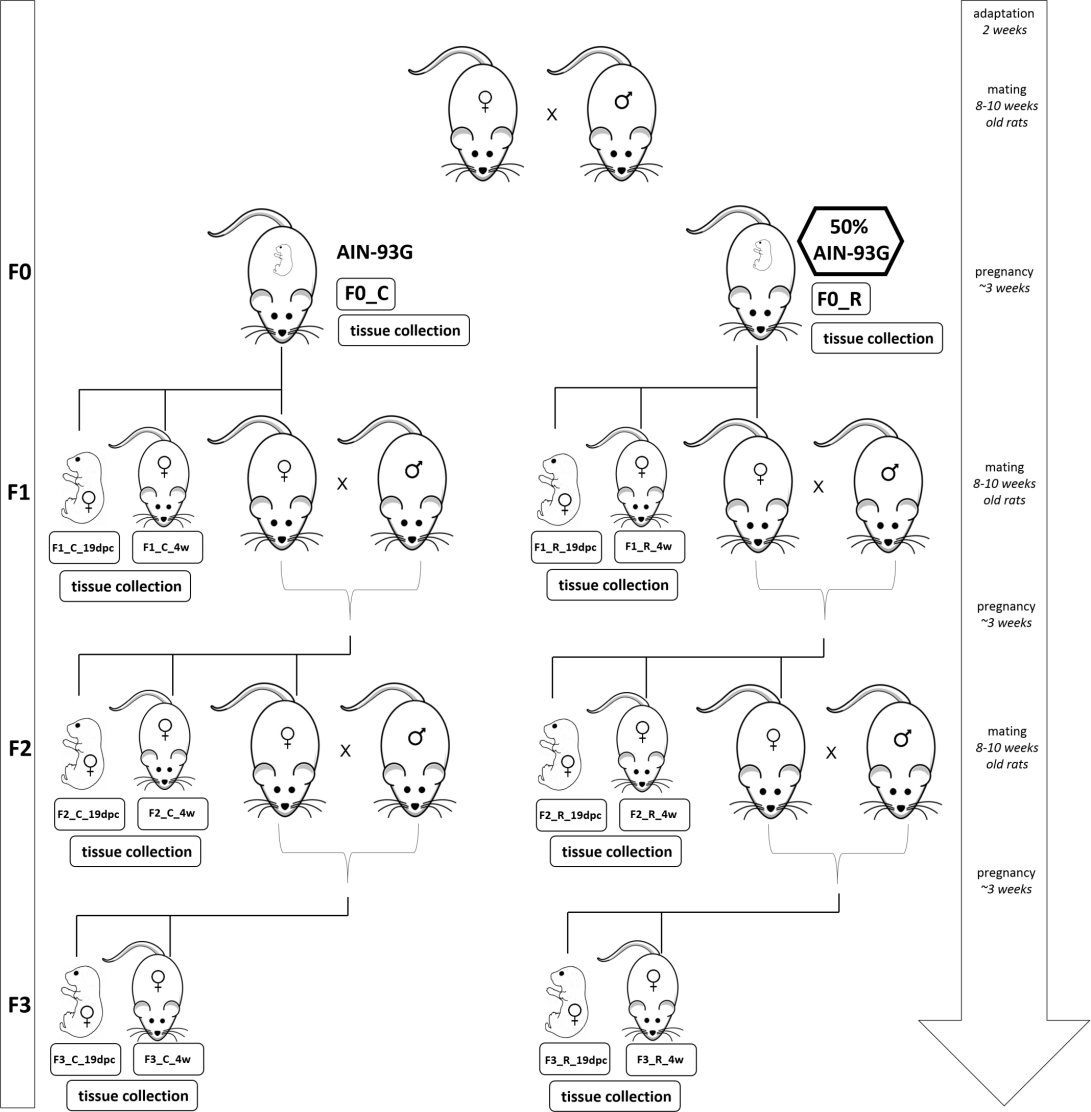
Study design showing four generations of rats. The experimental diet (50% of AIN-93G) was applied only in pregnant dams from F0. After delivery, and until the end of experiment, all animal groups were fed standard AIN-93G diet *ad libitum*. F0-F3 –number of generation; C or R–control or restricted group; 19dpc–fetuses in 19^th^ day of pregnancy; 4w –animals at the age of 4 weeks.

All experimental procedures were carried out in compliance with the international principles for laboratory animals, and the study protocol was approved by a Bioethical Commission for Animal Care and Use in Poznan, Poland (approval no. 37/2014). Wistar rats aged 10 weeks were purchased from Charles River Laboratories (Germany). The rats were housed in individual cages on a 12 h light–dark cycle (light on from 8 a.m to 8 p.m) at a temperature of 20 ± 1°C. Following the acclimatization period, 34 virgin female rats were mated with 15 males. During the mating phase, the animals were allowed to eat standard AIN-93G diet *ad libitum* (Table A in S1 File) [14]. Successful mating was confirmed by the presence of a vaginal plug, and the female rats were then assigned to a control diet (C group) or a restricted (R group) diet. The control diet was the AIN-93G diet given *ad libitum*. The R group was fed 50% of the typical food intake of the C group, with a correction for body mass. This feeding scheme resulted not only in calorie deficiency, but also in deficiencies of each nutrient in the F0_R group. After delivery, the litter size was recorded and, three days after delivery, the litters were culled to a maximum of eight pups to minimize variation in nutrition during the suckling period. Following parturition, all rats were introduced to the AIN-93G diet *ad libitum* and continued to receive this diet for the remainder of the experimental protocol. The rats were weaned at the age of 4 weeks; some of the females were then anesthetized and sacrificed for tissue collection, while the remaining females were kept alive and used for mating, which occurred when the rats were aged 8–10 weeks. Until then, the animals of the same litter were grouped two per cage. The same procedure was continued to obtain F2 and F3 generations where each time 15 males were used for mating. During the gestational period, food intake was monitored daily and body weight was measured weekly using an electronic scale. Additionally, six pregnant dams of the F0, F1, and F2 generations were randomly selected on day 19 of pregnancy (19 dpc) and sacrificed. The animals were fasted overnight, anesthetized by CO_2_ inhalation, and euthanized by cardiac puncture. Fetal liver samples were taken, immediately frozen in liquid nitrogen, and stored at -80°C for further analysis. The full design of the experiment is presented in Fig 1. Since in the whole experiment only females were studied, the molecular sex of the fetuses was established by PCR amplification of the Y derived gene (*Uty*—*Ubiquitously Transcribed Tetratricopeptide Repeat Containing*, *Y-Linked*). The DNA was isolated with phenol:chloroform:isoamyl alcohol acid mixture 24:25:1 (Sigma) following PCR using standard conditions on a Biometra thermocycler. The primers for the *Uty* gene were as follows: F: 5’ gatcaacaacgtccgtttca and R: 5’ gaactggctgaagagggtga. The amplicon of 503 bp was checked on agarose gel by electrophoresis in a presence of DNA from an adult male, female and a negative control (sample without DNA).

### Real-time PCR

The relative transcript level was measured for the genes involved in DNA methylation and histone modification pathways, namely: *Dnmt1* (*DNA Methyltransferase 1*), *Dnmt3a* (DNA *Methyltransferase 3 Alpha*), *Dnmt3b* (*DNA Methyltransferase 3 Beta*), *Mecp2* (*Methyl–CpG Binding Protein 2*), *Hdac1* (*Histone Deacetylase 1*), *Sin3a* (*SIN Transcription Regulator Family Member A*). The total RNA was extracted from the liver, femoral muscle, and visceral white adipose tissues using Trizol (Roche) reagent and following the standard protocol. The quality and quantity of the RNA isolates were checked on a Nanodrop system. cDNA synthesis was performed using 1 μg of RNA and iScript cDNA Synthesis Kit (Bio-Rad). Real-time PCR was conducted on a Light Cycler 480 Instrument (Roche) using the SYBR Green detection format (Roche). The analysis was performed in duplicate for each sample (six females in each generation/age/diet group). We used two genes as internal control: *Hprt* (*hypoxanthine-guanine phosphoribosyltransferase gene*) and *Tbp* (*TATA box binding protein gene*). All details concerning primers and amplicon length are shown in Table B in S1 File. Relative transcript quantification was performed using the 2^-ΔΔ*C*T^ method, following Livak and Schmittgen [15].

### Total H3 acetylation measurements

The histone proteins were extracted from the liver tissue using a Histone Extraction kit (Abcam), following the manufacturer’s protocol. The quantity of obtained extract was checked on a Qubit fluorimeter (Thermo Fisher Scientific) using a Qubit Protein Assay Kit (Thermo Fisher Scientific). The total histone H3 acetylation level was measured by colorimetric reaction with the use of a Histone H3 Total Acetylation Detection Fast Kit (Abcam). Briefly, 150 ng of histone proteins were applied to 8-well strips coated with specific anti-acetyl histone H3 antibody. The standard curve was prepared as a 1:1 dilution series (from 100 ng to 1.562 ng and a negative control). The samples were incubated in wells with antibody buffer for 1.5 h at room temperature. Afterwards, the wells were washed three times with 1× wash buffer. Next, the captured acetyl histone H3 was washed with detection antibody at a 1:1000 dilution for 1 h at room temperature on an orbital shaker at 100 rpm, following six washes with a 1× wash buffer. The color development reagent was then added for 5 min. Next the color reaction was stopped by adding stop solution buffer. Within next 5 minutes the intensity of absorbency (450nm) was measured on a Synergy2 device (Biotek). The analysis was performed in duplicate on female fetuses at day 19 of pregnancy in F1, F2 and F3 generations and in four-week-old animals from F1. Each group was composed of six animals. The results are shown as a mean absorbency ± S.D. in the C and R groups.

### S-adenosylmethionine and S-adenosylhomocysteine measurements

Measurements of liver S-adenosylmethionine (SAM) and S-adenosylhomocysteine (SAH) concentrations were performed in triplicate, according to the methods described by She et al. [16] and Wang et al. [17], using high-performance liquid chromatography (HPLC). Liquid nitrogen was poured on frozen tissue samples, which were then homogenized using a chilled mortar and pestle. Around 0.1 g of homogenized liver samples were placed in Eppendorf tubes and weighed. Five volumes of 0.4 M HClO_4_ were added to each sample. The homogenate was centrifuged at 10,000×g for 20 minutes in 4°C, and each supernatant was filtered through a 0.45 μm membrane. 25 μl of the extracts were applied to a TSK gel ODS-80T_M_ (25×4.6mm) column (Tosoh). SAM and SAH were eluted under gradient conditions. Solvent A consisted of 8 mM heptanosulphonic acid and 40 mM NH_4_H_2_PO_4_ adjusted to pH 3.0 with HCl, and solvent B was 100% methanol. The HPLC column was equilibrated with 82% solvent A and 18% solvent B. The gradient was 8 minutes to increase solvent B to 38%, and then 12 minutes at the new condition. The flow rate was 1 ml/min, and the absorbance was monitored at 254 nm. HPLC was performed at 35°C. SAM and SAH were identified according to their retention times and cochromatography with SAM and SAH standards (Sigma-Aldrich, Poland). The analysis was performed for F0 dams as well as four-week-old progeny from F1, F2, and F3 generations. In each group, 6 animals were analyzed in triplicate. Quantification was based on the integration of peak heights and comparison to the standard calibration curves of SAM and SAH.

### Statistical analysis

The results are presented as means with standard errors. The differences between the C and R groups were assessed using Student’s *t*-test. Correlations were evaluated for statistical significance using Pearson’s test. P < 0.05 was taken to be statistically significant.

## Results

### The impact of maternal restricted diet on transcript level of the selected genes through generations

We analyzed the transcript levels of the key genes involved directly in the DNA methylation process, such as the methyltransferases (*Dnmt1*, *Dnmt3a*, *Dnmt3b*) and a gene coding a methyl CpG-binding protein associated with this machinery (*Mecp2*). The mRNA levels were also determined for two genes that play a crucial role in the histone modification pathway (*Hdac1* and *Sin3a*). The analysis was performed in duplicate for each animal. In the F0 mothers and the four-week-old F1 progeny, mRNA levels were measured in three tissues: the liver, muscle, and adipose. No significant differences were observed in the adipose tissue for any of the analyzed genes (Figures A and B in S1 File). Similar results were obtained for muscle (Figures C and D in S1 File), with the exception of the *Mecp2* gene in the four-week-old progeny (P = 0.042), where the transcript level was reduced in the restricted group. Since the adipose and muscle tissues were only weakly affected by the diet in the first generation, they were not studied in the F2 and F3 generations.

**Fig 2:**
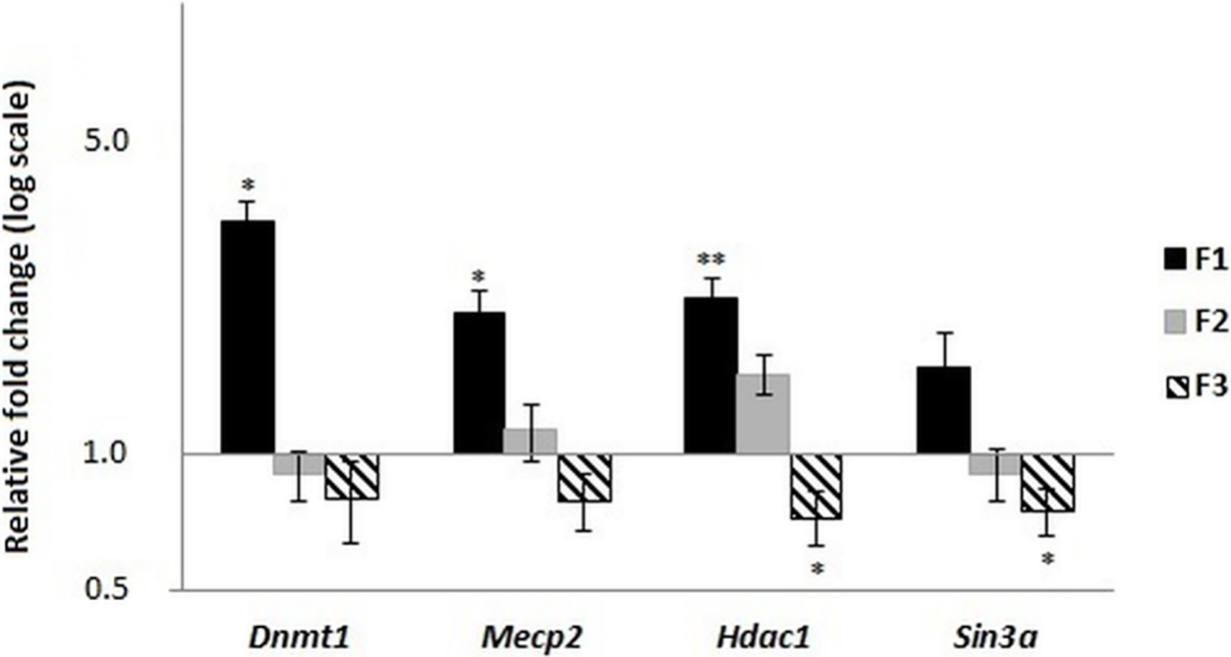
Expression of *Dnmt1*, *Mecp2*, *Hdac1*, and *Sin3a* genes in fetal livers (female fetuses aged 19 dpc) from F1, F2, and F3 generations. Results are shown as a fold change in restricted group in relation to controls (means ± SE; n = 6 per group). Asterisks indicate a significant difference between the respective C and R groups: * P < 0.05, ** P < 0.01.

**Fig 3:**
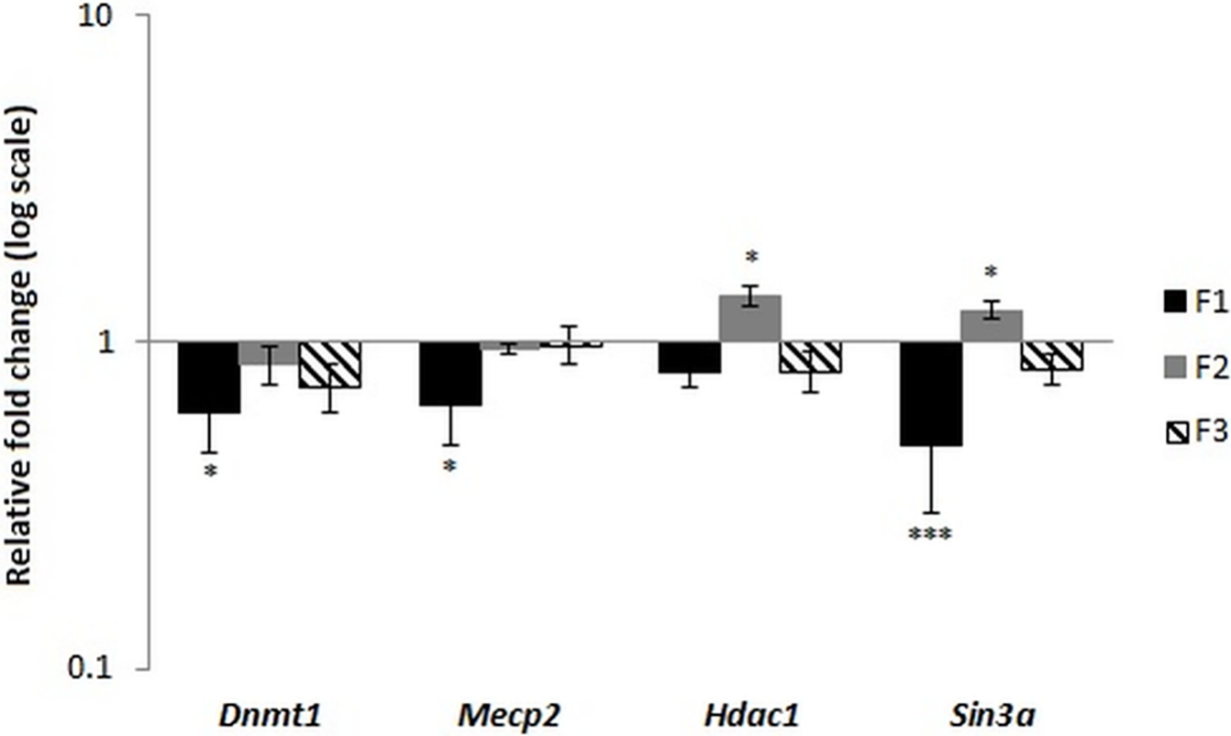
Expression of *Dnmt1*, *Mecp2*, *Hdac1*, and *Sin3a* genes in the livers of four-weeks old animals from generations F1, F2, and F3. Results are shown as a fold change in restricted group in relation to controls (means ± SE; n = 6 per group). Asterisks indicate a significant difference between the respective C and R groups: * P < 0.05, *** P < 0.001.

In contrast, the liver proved to be most susceptible to dietary manipulations, and transcript level changes were found in this tissue for all genes except for the *de novo* methyltransferases (*Dnmt3a* and *Dnmt3b*) studied in F1 fetuses (no differences between C and R groups), which were not analyzed in subsequent generations. We observed significant differences for the *Dnmt1*, *Mecp2*, *Hdac1*, and *Sin3a* genes between the control and restricted groups in the F1 generation. In details, the *Dnmt1* and *Mecp2* were upregulated in restricted group in fetal liver, while in older animals their mRNA level was reduced (Fig 2). The transcript level of *Hdac1* was significantly increased in fetuses from restricted group, while the *Sin3a* was decreased in four-weeks old progeny (Fig 3). The observed differences in F1 justified the examination of the mRNA levels in F2 and F3, where the transcript levels for *Dnmt1* and *Mecp2* were no longer significantly affected, but the mRNA levels for *Hdac1* and *Sin3a* genes maintain significant differences–*Hdac1* and *Sin3a* increased in F2 four-weeks-old animals, while in F3 both genes were downregulated in the fetal liver (Figs 2 and 3). This indicates that the changes programmed in the F1 animals during prenatal development in the genes involved in the histone modification pathway were inherited by the next generations.

Given that the genes being examined are functionally related through their biological pathways, we checked for correlations between their mRNA levels in the liver. In terms of fetal samples, it was seen that the transcript levels of *Dnmt3a*, *Dnmt3b*, *Mecp2*, and *Sin3a* were highly positively correlated within the F1_C_19dpc group (with correlation coefficients ranging from r^2^ = 0.83 to r^2^ = 0.98). In the F1_R_19dpc expression levels of all the analyzed genes were correlated with each other (correlation coefficients ranged from r^2^ = 0.83 to r^2^ = 0.99; Table 1). In the F2 generation, the mRNA levels of *Dnmt1*, *Mecp2*, *Hdac1*, and *Sin3a* were correlated within both F2_C_19dpc (ranging from r^2^ = 0.66, P = 0.0477 to r^2^ = 0.91, P = 0.0031) and F2_R_19dpc (ranging from r^2^ = 0.83, P = 0.0115 to r^2^ = 0.97, P = 0.0004) groups, except for the *Hdac1* gene in the restricted group (Table C in S1 File). In the F3 generation, only a few correlations were observed. In the F3_C_19dpc group, correlations were found for the *Dnmt1* and *Mecp2* genes (r^2^ = 0.72; P = 0.0322) and for the *Hdac1* and *Sin3a* genes (r^2^ = 0.79; P = 0.0174). In the F3_R_19dpc fetuses, the *Mecp2* transcription level correlated strongly with *Dnmt1* (r^2^ = 0.94; P = 0.0015) and *Hdac1* (r^2^ = 0.73; P = 0.0299)–Table D in S1 File. In the four-week-old progeny, correlations between transcript levels were observed only in the control groups: in F1_C_4w, *Hdac1* correlated with *Mecp2* (r^2^ = 0.98; P = 0.0001) and *Sin3a* (r^2^ = 0.74; P = 0.0274). In the F2 generation (F2_C_4w), *Hdac1* correlated with *Mecp2* (r^2^ = 0.89; P = 0.0050), while in F3 (F3_C_4w), *Dnmt1* correlated with *Mecp2* (r^2^ = 0.72; P = 0.0317) and *Sin3a* (r^2^ = 0.86; P = 0.0080).

**Table 1:** Correlation analysis of hepatic mRNA and histone H3 acetylation levels in F1 female fetuses (n = 6 per group).

	*Dnmt1*	*Mecp2*	*Hdac1*	*Sin3a*	*Dnmt3a*	*Dnmt3b*	Histone H3 acetylation level
F1_C_19dpc							
*Dnmt1*	1.00						
*Mecp2*	0.73 (NS)	1.00					
*Hdac1*	0.09 (NS)	-0.27 (NS)	1.00				
*Sin3a*	0.57 (NS)	0.83 (0.0402)	-0.01 (NS)	1.00			
*Dnmt3a*	0.77 (NS)	0.96 (0.0022)	-0.16 (NS)	0.85 (0.0298)	1.00		
*Dnmt3b*	0.80 (NS)	0.98 (0.0004)	-0.18 (NS)	0.88 (0.0199)	0.98 (0.0004)	1.00	
Histone H3 acetylation level	0.20 (NS)	0.60 (NS)	-0.48 (NS)	0.63 (NS)	0.42 (NS)	0.55 (NS)	1.00
F1_R_19dpc							
*Dnmt1*	1.00						
*Mecp2*	0.94 (0.0052)	1.00					
*Hdac1*	0.92 (0.0096)	0.86 (0.0278)	1.00				
*Sin3a*	0.96 (0.0020)	0.86 (0.0299)	0.96 (0.0025)	1.00			
*Dnmt3a*	0.94 (0.0051)	0.95 (0.0037)	0.99 (0.0001)	0.95 (0.0037)	1.00		
*Dnmt3b*	0.92 (0.0090)	0.79 (NS)	0.84 (0.0353)	0.94 (0.0058)	0.83 (0.0417)	1.00	
Histone H3 acetylation level	0.87 (0.0237)	0.70 (NS)	0.92 (0.0082)	0.95 (0.0030)	0.88 (0.0193)	0.85 (0.0297)	1.00

Significance levels are given in parentheses (NS: nonsignificant).

### Changes in H3 acetylation profile in liver of F1 fetuses

**Fig 4:**
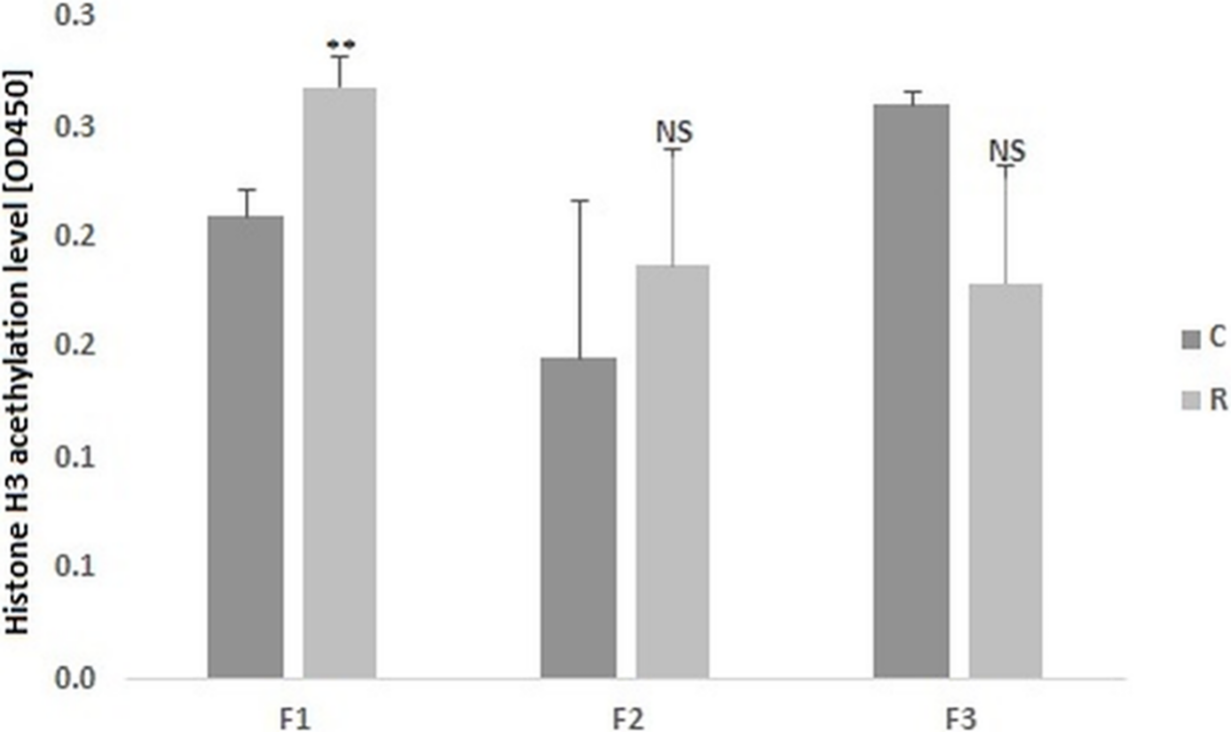
The differences in H3 acetylation level in fetal livers (female fetuses aged 19 dpc) from F1, F2, and F3 generations. Asterisk indicate a significant difference between the respective C and R groups: ** P < 0.01; NS–not significant. Data presented as the means ± SE (n = 6 per group).

The total hepatic histone 3 acetylation was measured in duplicates in a group of female fetuses aged 19 dpc from the F1, F2, and F3 generations as well as in four-week-old female animals from F1. We found that the F1_R_19dpc group had increased levels (by 28%) of total acetylation compared to F1_C_19dpc, and that this difference was statistically significant (P = 0.009). The increase by 55% and decreased by 18% of H3 acetylation were also observed in the F2 and F3 generations, respectively; however, the results were not significant, most likely due to the high standard deviation (Fig 4). Due to the fact that only in F1 fetuses the differences were significant we also controlled if in older animals (four-week-old) from F1 this alteration is maintain. The obtained results showed that there was no difference in H3 acetylation between the studied groups (increase by 4% in restricted group)–Figure E in S1 File. We also studied correlation between the histone H3 acetylation and the mRNA levels of *Hdac1* and *Sin3a* genes. A high such correlation was found in the F1_R_19dpc group, with a coefficient of r^2^ = 0.92 (P = 0.0082) for *Hdac1* and r^2^ = 0.95 (P = 0.0030) for *Sin3a*; this was nonsignificant in the controls (Table 1). In subsequent generations, the only correlation was found in the F3_C_19dpc group for the *Sin3a* gene (r^2^ = 0.90; P = 0.0152).

### No effect of restricted diet on SAM and SAH levels in the liver

The levels of SAM and SAH and the ratio of SAM to SAH (which reflects the cellular methylation potential) were measured in triplicate in F0 dams, as well as in four-week-old progeny from three generations. In general, there were no differences between the progeny (Figure F in S1 File). The only significant change was seen in the SAH level of F0 dams from the restricted group, which decreased by almost 20% (P = 0.018). We found no correlation in any generation between the SAM-to-SAH ratio and hepatic transcript levels.

## Discussion

The impact of restricted diet applied in this study was already evaluated in our previous research where the physiological parameters (lipid profile) as well as body weight, feed consumption, litter size and fetuses weight were analyzed. We found that in terms of lipid profile the greater impact of the restricted diet was found in dams (F0), where the total cholesterol, HDL and triglycerides decreased, while in F1 progeny at the age of four-weeks only LDL concentration was altered—increased in restriction group [4]. On the other hand, the body mass of the F0 dams as well as F1 fetuses from restricted groups were reduced by 12 and 20%, respectively. Here, we also analyzed body composition in four-week-old progeny from three studied generations. Such parameters as body mass, percentages of fat mass and lean body mass were not significantly changed in response to the studied diet (Table E in S1 File).

The main aim of this study, was the examination of the expression profile of genes involved in epigenetic processes in three tissues through four generations of rats: pregnant dams and their female progeny from the F1, F2, and F3 generations at ages of 19 dpc and 4 weeks. The F1 rats experienced prenatal restricted diet. We found that the adipose and muscle tissues were not sensitive to this feeding type, unlike liver tissue. This is in agreement with Zhang et al. [18] who studied the transcriptional changes in three tissues: liver, skeletal muscle and adipose and also observed that the liver, as the major metabolic organ, is more susceptible to nutritional manipulation. We expected that the restricted diet, acting on a developing fetus, would potentially induce a wide range of changes in the expression of genes involved in DNA methylation. Since the methylome is established during early prenatal development, we evaluated two crucial *de novo* methyltransferases genes (*Dnmt3a* and *Dnmt3b*). No differences were found in the fetal livers in terms of the transcript levels in response to the prenatal restricted diet. To the contrary, it was demonstrated that the murine expression of *Dnmt3a* and *Dnmt3b* was susceptible to changes induced by an early diet deficient in methyl donors [19, 20]. On the other hand, we observed in this study that two other genes associated with DNA methylation (*Dnmt1* and *Mecp2*) were susceptible to the studied diet. This effect was detected only in the first generation of the progeny and not in the F0 dams. Previous studies have shown that there is a correlation between the transcript level of *Dnmt1* and methylation status when different diets were applied. For example, a prenatal protein-restricted diet in rats led to a decrease in the hepatic *Dnmt1* mRNA level in progeny, resulting in promoter hypomethylation of the glucocorticoid receptor 1 (*GR1*) gene [21]. A choline-deficient diet during pregnancy in rats induced an increase in the liver *Dnmt1* transcript level in offspring, causing global hypermethylation [22]. Since we observed changes in *Dnmt1* and *Mecp2* transcripts upon prenatal restricted diet, it can be assumed that alternations in DNA methylation might occur. However, it must be kept in mind that the way from the transcript to functional protein involves different posttranscriptional and posttranslational modifications. Thus, also studies focusing on the protein level should be consider in a future.

It should be also taken into consideration that the DNA methylation machinery also depends on the availability of methyl donors [23]. In this study, we measured the SAM-to-SAH ratio, which is a widely accepted method for assessing cellular methylation potential [24]. It has been shown that a decreased SAM-to-SAH ratio in the liver and placenta of rats fed with folate-deficient diet corresponded to a reduction in global DNA methylation [25]. On the other hand, global DNA methylation in the livers of mice on a methionine-deficit diet correlated negatively with SAH level [26]. Nohara et al. [27] also found an increased global level of 5-methylcytosyne (5mC) in mice on a methyl-deficient diet, despite the decreased SAM level. In our study, the hepatic levels of SAM and SAH did not alter in the generations we studied. However, this finding does not exclude potential methylation changes induced by the restriction diet, since the *Dnmt1* and *Mecp2* transcripts underwent alteration. Similarly, Xia et al. [28] noticed that variation in DNA methylation levels in mouse livers is associated with the expression of *Dnmt*s. Taking the above into consideration, future studies of DNA methylation potential should be performed using different approaches such as the SAM-to-SAH ratio and methyltransferase gene expression analysis.

Unlike DNA methylation, histone modifications have not been as widely investigated in the context of nutritional modulations. Histone acetylation is considered an epigenetic mark related to transcriptional activation and is modulated by histone acetyltransferases (HAT) and deacetylases (HDAC). These enzymes have been classified into many families [29]. Here, we selected two genes: *Hdac1* and *Sin3a* encoding proteins, which are a part of the corepressor complex Sin3 belonging to the class I deacetylases [30]. We observed increased expression in the hepatic *Hdac1* gene in the fetuses, while *Sin3a* decreased in the 4-weeks old progeny in response to the restricted diet in animals from the first generation. *Hdac1* activity has been extensively investigated in the context of the restricted diet effect on longevity. An increase was observed in its expression, leading to deacetylation of important genes associated with ageing processes [31]. Surprisingly, in our study, in spite of the increased *Hdac1* mRNA levels, global histone H3 acetylation increased significantly in the F1 generation of the prenatally restricted rats, and the same trend was also observed in F2. Taking into consideration the complexity of the histone modification machinery, it should be keep in mind that we cannot expect a simple links between acetylation of H3 and *Sin3a* mRNA level. There are some other reports on dietary restriction that show increased histone acetylation. Kawakami et al. [32] analyzed site-specific histone acetylation (H3K9, K27, and K56) in the livers of rats fed this diet postnatally, and found a 20%–30% increase in acetylation. The enhanced acetylation of multiple cellular and nuclear proteins in the liver was also observed by Nakamura et al. [33]. In one of these studies, the activity of the selected NAD-dependent deacetylases (sirtuins) belonging to class III was evaluated, and similarly to our results the authors observed different patterns of their expression (increased or decreased). It seems that the relationship between the expression of deacetylases and histone acetylation is complex and depends on modification site, deacetylases classes, and target genes. We have shown that prenatal dietary restriction can induce changes in the global histone H3 acetylation level which were positively correlated with the transcript level of the studied genes. It was noticed that the correlations found in F1 fetuses were predominantly observed in the restricted group. In four-week-old animals (F1) as well as in fetuses from F2 and F3 the differences in H3 acetylation were not significant. It must also be considered that the genes responsible for DNA methylation and selected histone modifications act together influencing transcriptional activity [34]. We thus performed correlation analysis between the expression of all the studied genes in the control and the restricted groups. It was found that significantly more correlations were observed in animals from restricted diet, but only in fetuses from the F1 generation. Since the studied genes cooperate and play a role in transcription silencing [30], it can be anticipated that the restricted diet, when applied prenatally, induced stronger cooperation between genes than in the control group leading to potential epigenetic changes. Since the prenatal period is sensitive to various dietary manipulations, we expected a wide range of changes which, when programmed in the F1 progeny, can be potentially inherited by subsequent generations [35]. In our results only *Hdac1* and *Sin3a* genes showed differences in all generations. The F1 and F2 animals from the restricted groups had an increased level of *Hdac1*; in F3, the first generation free from the influence of the experimental factor, *Hdac1* transcription decreased. Significant changes in mRNA were also observed for the *Sin3a* gene, but these did not maintain the same direction over the three generations. For the genes involved in DNA methylation (*Dnmt1* and *Mecp2*), such transgenerational changes were not found. Since we observed that mRNA levels changed over three generation mainly for histone modification genes, it can be assumed that this epigenetic process could be more sensitive to dietary factors. There are limited data on the impact of the different deficient diets on several generations. In the study of Hoile et al. [36], in which a protein-restricted diet was applied during pregnancy, transcriptional analysis was performed for three generations of progeny. The authors found that the number of differently expressed genes varied from 1680 in F1 to 2062 in F3. Of these, only approximately 100 showed differences in all three generations, but only a small group had the same trend of expression (up or down regulation in all three generations). Radford et al. [37] examined the influence of maternal undernutrition in the last week of mouse gestation on gene expression in progeny. They found that the imprinted genes that play a unique role in epigenetic dosage control were not sensitive to nutritional restriction in the F1 or F2 generation, while a significant increase was observed in transcript levels for genes involved in lipid and fatty acid metabolism. Taking the above into consideration, it seems that, although the influence of different diets on gene expression in the F1 generation is significant, only a small number of genes maintain the programmed changes in the subsequent generations.

## Conclusions

This paper describes the impact of a prenatal restricted diet on four generations of rats. We have shown that the expression of key genes involved in the two main epigenetic mechanisms (DNA methylation and histone modifications) were significantly altered in the F1 generation, while these changes were not as obvious in the F2 and F3 generations. Moreover, the dietary restriction induced global histone H3 acetylation changes, with the most prominent effect detected in F1. Our study indicated that applied a prenatal restriction diet may induce long-term effects on the epigenome across generations. Future studies are needed to better understand the gene–nutrition interactions.

## Supporting information

S1 File Supporting information file. (DOC)
