# Supplementary material for: Transgenerational effects of prenatal restricted diet on gene expression and histone modifications in the rat
Source: PLoS One. 2018 Feb 23;13(2):e0193464. doi: 10.1371/journal.pone.0193464 (PMC5825138; doi:10.1371/journal.pone.0193464)

**S1 file**

**Supporting information file**

**Transgenerational effects of prenatal restricted diet on gene expression and histone modifications in the rat**

Joanna Nowacka-Woszuk,^1*^ Izabela Szczerbal,^1^ Anna M. Malinowska,^2^ Agata Chmurzynska^2^

^1^Department of Genetics and Animal Breeding, Poznan University of Life Sciences, Wolynska 33, 60-637 Poznan, Poland

^2^Institute of Human Nutrition and Dietetics, Poznan University of Life Sciences, Wojska Polskiego 31, 60-624 Poznan, Poland

^*^Corresponding author’s e-mail address: [jnowacka@up.poznan.pl](mailto:jnowacka@up.poznan.pl)

Table A. The composition of the AIN-93G diet.

| **Ingredient** | **g/kg diet** |
| --- | --- |
| Cornstarch | 397.486 |
| Casein | 200.000 |
| Dextrinized cornstarch | 132.000 |
| Sucrose | 100.000 |
| Soybean oil | 70.000 |
| Fiber | 50.000 |
| Mineral mix (AIN-93G)^a^ | 35.000 |
| Vitamin mix (AIN-93G)^b^ | 10.000 |
| L-cystine | 3.000 |
| Choline bitartrate | 2.500 |
| Tert-butylhydroquinone | 0.014 |

^a^Mineral mix (composition in mg): calcium 5000, phosphorus 1561, potassium 3600, sulfur 300, sodium 1019, chloride 1571, magnesium 507, iron 35, zinc 30, manganese 10, copper 6.0, iodine 0.2, molybdenum 0.15, selenium 0.15.

^b^Vitamin mix (composition in mg, except fat soluble vitamins): nicotinic acid 30, pantothenate 15, pyridoxine 6, thiamin 5, riboflavin 6, folic acid 2, D-biotin 0.200, vitamin K 0.750, vitamin B12 0.025, and vitamin A 4000U/kg diet, vitamin D3 1000U/kg diet, vitamin E 75 U/kg diet.

Table B. Real-time PCR primers and amplicon length of the studied genes.

| Gene | Primer sequences | Amplicon length | Reference |
| --- | --- | --- | --- |
| *Dnmt1* | F: 5’ GGTTCTGCGCGGGGACAGAC  R: 5’ CCGGCAACATGGCCTCAGGG | 183 bp | Xu et al., 2015 |
| *Mecp2* | F: 5’ GGACGCGAAAGCTTAAACAG  R: 5’ AGGAGGTGTCTCCCACCTTT | 138 bp | this study |
| *Hdac1* | F: 5’ CCGGCTTCTGTTACGTCAAT  R: 5’ CATGACCCGGTCTGTGGTAT | 140 bp | this study |
| *Sin3a* | F: 5’ AGGTGGCTCGTCTCTTCAAA  R: 5’ GTGCCTCCATGGTCATTTCT | 139 bp | this study |
| *Dnmt3a* | F: 5’ ACGCCAAAGAAGTGTCTGCT  R: 5’ CTTGGCTATTCTGCCGTGTT | 137 bp | this study |
| *Dnmt3b* | F: 5’ TCTGGCCACCTTCAATAAGC  R: 5’ TGGTCCTCCAGTGAGTCTCC | 120 bp | this study |
| *Hprt* | F: 5’ CAGTCAACGGGGGACATAAAAG  R: 5’ ATTTTGGGGCTGTACTGCTTGA | 146 bp | Nowacka-Woszuk et al., 2015 |
| *Tbp* | F: 5’ ATCCTTCACCAATGACTCCTATG  R: 5’ ATGATGACTGCAGCAAACC | 190 bp |

Xu HX, Qin JZ, Zhang KY, Zeng WX. Dynamic expression profile of DNA methyltransferases in rat testis development. Pol J Vet Sci. 2015; 18(3): 549-556.

Nowacka-Woszuk J, Pruszynska-Oszmalek E, Szydlowski M, Sadkowski S, Szczerbal I. Diet-induced variability of the resistin gene (Retn) transcript level and methylation profile in rats. BMC Genet. 2015; 16: 113.

Table C. The correlation analysis of the hepatic mRNA levels and H3 acetylation level in F2 fetuses (n=6 per group).

|  | *Dnmt1* | *Mecp2* | *Hdac1* | *Sin3a* | Histone H3 acetylation level |
| --- | --- | --- | --- | --- | --- |
| F2_C_19dpc | | | | | |
| *Dnmt1* | 1.00 |  |  |  |  |
| *Mecp2* | 0.66  (0.0477) | 1.00 |  |  |  |
| *Hdac1* | 0.74  (0.0271) | 0.74  (0.0289) | 1.00 |  |  |
| *Sin3a* | 0.91  (0.0031) | 0.61  (NS) | 0.83  (0.0121) | 1.00 |  |
| Histone H3 acetylation level | 0.13  (NS) | 0.02  (NS) | 0.40  (NS) | 0.30  (NS) | 1.00 |
| F2_R_19dpc | | | | | |
| *Dnmt1* | 1.00 |  |  |  |  |
| *Mecp2* | 0.93  (0.0018) | 1.00 |  |  |  |
| *Hdac1* | 0.08  (NS) | 0.04  (NS) | 1.00 |  |  |
| *Sin3a* | 0.97  (0.0004) | 0.83  (0.0115) | 0.09  (NS) | 1.00 |  |
| Histone H3 acetylation level | 0.11  (NS) | 0.09  (NS) | 0.01  (NS) | 0.08  (NS) | 1.00 |

Significance levels are given in parentheses (NS: nonsignificant).

Table D. The correlation analysis of the hepatic mRNA levels and H3 acetylation level in F3 fetuses (n=6 per group).

|  | *Dnmt1* | *Mecp2* | *Hdac1* | *Sin3a* | Histone H3 acetylation level |
| --- | --- | --- | --- | --- | --- |
| F3_C_19dpc | | | | | |
| *Dnmt1* | 1.00 |  |  |  |  |
| *Mecp2* | 0.72  (0.0322) | 1.00 |  |  |  |
| *Hdac1* | 0.01  (NS) | 0.01  (NS) | 1.00 |  |  |
| *Sin3a* | 0.04  (NS) | 0.05  (NS) | 0.79  (0.0175) | 1.00 |  |
| Histone H3 acetylation level | 0.36  (NS) | 0.31  (NS) | 0.56  (NS) | 0.81  (0.0152) | 1.00 |
| F3_R_19dpc | | | | | |
| *Dnmt1* | 1.00 |  |  |  |  |
| *Mecp2* | 0.94  (0.0015) | 1.00 |  |  |  |
| *Hdac1* | 0.58  (NS) | 0.73  (0.0299) | 1.00 |  |  |
| *Sin3a* | 0.01  (NS) | 0.05  (NS) | 0.25  (NS) | 1.00 |  |
| Histone H3 acetylation level | 0.51  (NS) | 0.30  (NS) | 0.11  (NS) | 0.02  (NS) | 1.00 |

Significance levels are given in parentheses (NS: nonsignificant).

Table E. The body composition of the four-week old animals (means ± SD; n=6 per group).

| **Parameter** | **C group** | **R group** | **P value** | **Reference** |
| --- | --- | --- | --- | --- |
| **F1** | | | | Nowacka-Woszuk et al., 2017 |
| Body mass [g] | 64.6 ± 1.8 | 61.4 ± 3.7 | 0.46 |
| Fat mass [g] | 5.6 ± 0.8 | 5.0 ± 1.2 | 0.73 |
| Fat mass [%] | 8.5 ± 1.1 | 7.8 ± 1.3 | 0.71 |
| Lean body mass [g] | 50.5 ± 1.2 | 48.2 ± 2.2 | 0.37 |
| Lean body mass [%] | 78.2 ± 0.4 | 78.8 ± 1.1 | 0.65 |
| **F2** | | | | this study |
| Body mass [g] | 59.4 ± 3.6 | 57.2 ± 1.8 | 0.60 |
| Fat mass [g] | 4.1 ± 0.8 | 3.2 ± 0.5 | 0.37 |
| Fat mass [%] | 6.8 ± 1.2 | 5.5 ± 0.7 | 0.38 |
| Lean body mass [g] | 47.2 ± 1.2 | 45.8 ± 1.2 | 0.66 |
| Lean body mass [%] | 79.4 ± 0.5 | 80.1 ± 0.5 | 0.43 |
| **F3** | | | |
| Body mass [g] | 60.3 ± 2.6 | 63.5 ± 2.8 | 0.90 |
| Fat mass [g] | 4.7 ± 0.8 | 5.5 ± 0.9 | 0.70 |
| Fat mass [%] | 7.6 ± 1.0 | 8.5 ± 1.7 | 0.70 |
| Lean body mass [g] | 46.8 ± 2.2 | 49.9 ± 2.0 | 0.84 |
| Lean body mass [%] | 77.7 ± 0.8 | 78.6 ± 0.9 | 0.91 |

Nowacka-Woszuk J, Madeja ZE, Chmurzynska A. Prenatal caloric restriction alters lipid metabolism but not hepatic Fasn gene expression and methylation profiles in rats. BMC Genet. 2017 Aug 15;18(1):78.

Figure A. The expression of *Dnmt1, Mecp2, Hdac1,* and Sin3a genes in adipose tissue in F0 dams. Results are shown as a fold change in restricted group in relation to controls (means ± SE; n=6 per group).


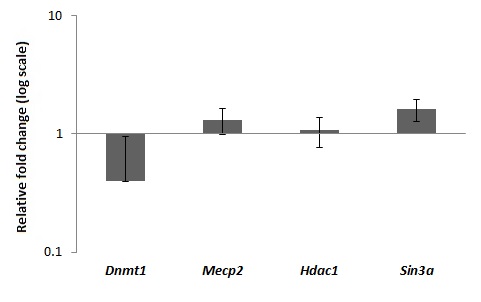


Figure B. The expression of *Dnmt1, Mecp2, Hdac1,* and *Sin3a* genes in adipose tissue in F1 four-week-old progeny from R group. Results are shown as a fold change in restricted group in relation to controls (means ± SE; n=6 per group).


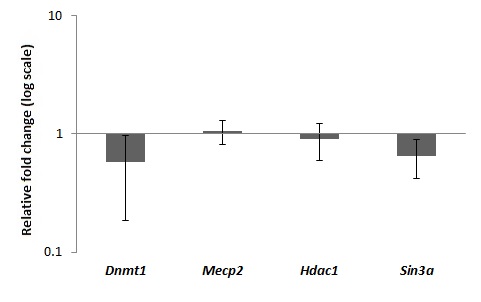


Figure C. The expression of *Dnmt1, Mecp2, Hdac1,* and *Sin3a* genes in muscle tissue in F0 dams from R group. Results are shown as a fold change in restricted group in relation to controls (means ± SE; n=6 per group).


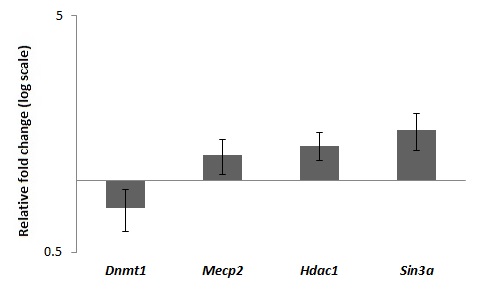


Figure D. The expression of *Dnmt1, Mecp2, Hdac1,* and *Sin3a* genes in muscle tissue in F1 four-week-old progeny from R group. Results are shown as a fold change in restricted group in relation to controls (means ± SE; n=6 per group). Asterisks indicate a significant difference between the respective C and R group, * P < 0.05.


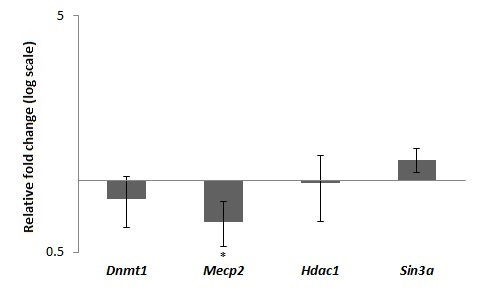


Figure E. The differences in H3 acetylation level in livers of female progeny four-week-old (F1 generation). NS – nonsignificant difference between groups. Values are expressed as the means ± SE (n=6 per group).


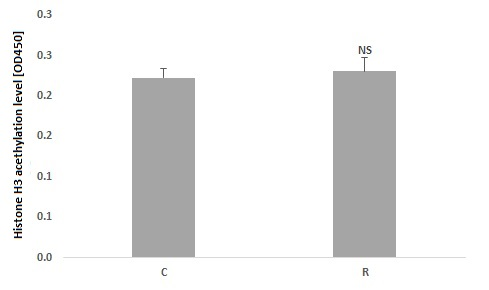


Figure F. The SAM to SAH ratio in F0 dams and four-week-old progeny from F1, F2, and F3 generations. Values are expressed as the means ± SE (n=6 per group). The differences between C and R groups in each generation were nonsignificant.


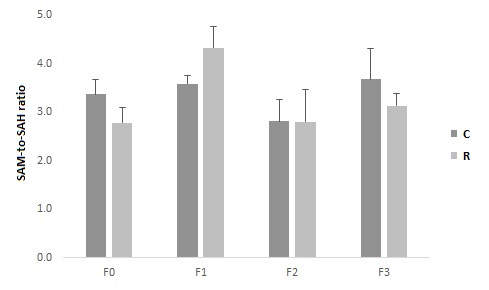

Supplement: S1 File — (DOC) [file pone.0193464.s001.doc]
